# Long Used but Hardly Known: Synthesis and Crystal Structure of Tritium Breeding Li_2_Be_2_O_3_

**DOI:** 10.1002/chem.202502209

**Published:** 2025-08-11

**Authors:** Georg Krach, Jennifer Steinadler, Robert Calaminus, Bettina V. Lotsch, Wolfgang Schnick

**Affiliations:** ^1^ Department of Chemistry University of Munich (LMU) Butenandtstraße 5–13 Munich 81377 Germany; ^2^ Max Planck Institute for Solid State Research Heisenbergstraße 1 Stuttgart 70569 Germany

**Keywords:** beryllium, fusion reactor, solid‐state structures, tritium breeder

## Abstract

A main challenge for the operation of a nuclear fusion reactor is the consumption of tritium during the fusion process and the limited availability of tritium in natural resources or its production in nuclear power plants. The most promising approach is breeding of new tritium within the operating fusion reactor. For this purpose, suitable breeding materials are needed. Lithium beryllium oxides are a promising class of compounds, as they unite both target and neutron multiplier in one material. While there have already been studies on sintered ceramics in the Li_2_O·BeO system, the crystal structure of compounds of a defined composition has so far remained unsolved. Herein, we report on the synthesis of phase‐pure Li_2_Be_2_O_3_ in a high‐temperature (HT) approach and its structure determination by single‐crystal X‐ray diffraction (sc‐XRD). In addition, the compound was characterized by powder X‐ray diffraction (PXRD), solid‐state nuclear magnetic resonance (NMR) spectroscopy, and elemental analysis. The thermal stability, which is important for use as blanket material in a fusion reactor, was examined with differential scanning calorimetry (DSC).

## Graphical abstract

The ternary oxide Li_2_Be_2_O_3_ was synthesized in a HT approach starting from Li_2_O and BeO using a radiofrequency furnace. Although, the compound has been known for almost 60 years, its crystal structure has only now been elucidated and a phase‐pure synthesis makes the determination of directly assignable properties possible. Li_2_Be_2_O_3_ is discussed for its usage in fusion reactors, as it breeds essential tritium under neutron irradiation.

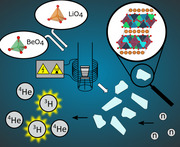

## 1 Introduction

Turning away from energy generation by burning fossil fuels is a key challenge of our time. In addition to the expansion of renewable energies such as photovoltaics or wind power, researchers have been working for almost 80 years on making the fusion reaction between deuterium (^2^H) and tritium (^3^H) controllable in a reactor and usable for energy generation (Equation 1).^[^ ^1^ ^]^

(1)

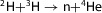




A major challenge in research for building a fusion reactor is the demand for tritium. While the amount of deuterium required for this process can be found in inexhaustible quantities in water, tritium is quite rare in natural resources. For the initial start of a fusion reactor, tritium can be bred, for example, in a heavy or pressurized water reactor. However, the amount of tritium required to operate a fusion reactor is too high to replenish it from these common sources. Hence, it has to be bred inside the fusion reactor, which is done by the reaction of a neutron with ^6^Li, forming an *α*‐particle and one ^3^H atom according to Equation 2.^[^ ^2^ ^]^

(2)

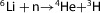




In theory, the neutron originating from the fusion of ^2^H and ^3^H (Equation 1), is sufficient to generate a new ^3^H atom through the reaction with ^6^Li (Equation 2). Under realistic conditions, however, this ratio is not sufficient, as the neutron can react with other parts of the reactor. Therefore, a suitable material like lead or beryllium is needed to multiply the number of neutrons according to an (n,2n) nuclear reaction (Equation 3).

(3)

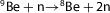




For this purpose, pebbles of elemental Be were tested in combination with Li‐containing ceramics. However, the formation of gaseous ^3^H and ^4^He under breeding conditions at 650 °C leads to a swelling of the Be metal up to 16% and thus exerts pressure on the ceramic, which can cause it to break.^[^ ^3^, ^4^ ^]^ Furthermore, the oxidation of elemental Be and the associated increasing brittleness is a safety issue. To circumvent this problem, other breeding materials like Be_12_Ti or concepts such as mixed, liquid molten fluoride salts of Li, Na, and Be were investigated.^[^ ^4^, ^5^, ^6^, ^7^ ^]^ Although such compounds outperform elemental Be in terms of swelling or oxidation resistance, they present other problems, such as corrosive behavior or the formation of reactive elemental fluorine or, in the case of Be_12_Ti, the need for an additional Li‐containing material.

Other potential materials are sintered ceramics made of Li_2_O and BeO.^[^ ^8^ ^]^ They combine the neutron multiplier ^9^Be and the breeding nucleus ^6^Li in one compound. The first synthesis was carried out in 1966 by *Turner* and *Bartram*, who reacted Li_2_CO_3_ with BeO and obtained colorless crystals with monoclinic metrics.^[^ ^9^ ^]^ From the chemical analysis, they derived the sum formula Li_2_Be_2_O_3_. The existence of this material and the metrics was confirmed by *Kastner* and *Hoppe* in 1975.^[^ ^10^ ^]^ They also found another compound with triclinic metrics, namely Li_4_BeO_3_. However, the structure of both materials remained undiscovered. In the following decades, Li_2_Be_2_O_3_ was part of several investigations regarding its thermal conductivity, tritium breeding rate, or optimized sintering conditions.^[^ ^8^, ^11^ ^]^ The compound class of lithium beryllium oxides was further extended, discussing Li_6_BeO_4_ as an ion conductor for (HT) lithium batteries with molten Li.^[^ ^12^ ^]^ At this point, it should be mentioned that the metrics of all compounds, Li_2_Be_2_O_3_, Li_4_BeO_3_, and Li_6_BeO_4_ were reported, but no information about the crystal structures, the phase‐pure syntheses, and thus directly assignable properties is known. Rather, the properties were determined on sintered samples that had the appropriate elemental composition. From today's perspective, the direct formulation of a sum formula seems daring, but it must be remembered that the analytical methods of the time were not yet as sophisticated as they are today. In addition, the low X‐ray contrast between Li and Be must be taken into account. However, understanding the structures is crucial to develop an understanding of the properties and to investigate the behavior under extreme conditions, such as those occurring in fusion reactors.

In this contribution, we report on the synthesis, structure elucidation, and properties of Li_2_Be_2_O_3_ in order to shed light on the chemistry of a compound that has been investigated for almost 60 years.

## 2 Results and Discussion

### 2.1 Synthesis

The title compound was synthesized starting from stoichiometric amounts of Li_2_O and BeO under HT conditions (Equation 4). An excess of 10% was used for Li_2_O.

(4) $$Li_{2}O+2\mathrm{BeO}\to Li_{2}Be_{2}O_{3}$$

**Figure 1:**
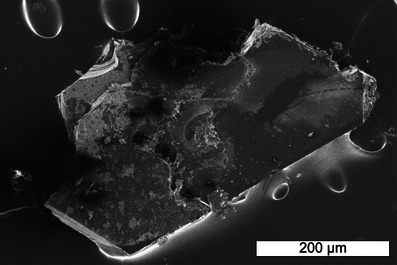
Exemplary SEM image of Li_2_Be_2_O_3_. Crystals are up to 400 µm in length and 200 µm in width.

The starting materials were finely ground, pressed to a pill, transferred to a tungsten crucible, and heated to 1500 °C under argon, followed by a sintering step at 1000 °C in a radiofrequency furnace. The colorless sample consists of crystals up to 400 µm in size (Figure 1), which are stable toward air and water, but not to diluted HCl. To remove residual Li_2_O, the sample was washed with distilled H_2_O. More information on the HT synthesis can be found in the Experimental Section.

### 2.2 Crystal Structure

The crystal structure of Li_2_Be_2_O_3_ was determined by single‐crystal X‐ray diffraction (sc‐XRD). The found monoclinic metrics (*C*2/*c* (no. 15), *a* = 8.546(2); *b* = 5.0222(14); *c* = 14.872(6) Å; *β* = 101.896(13)°; *Z* = 12)^[^ ^13^ ^]^ are similar to that found by *Turner* et al. and *Kastner* et al., but not identical.^[^ ^9^ ^]^ Additional crystallographic information can be found in Table S1–3.

**Figure 2:**
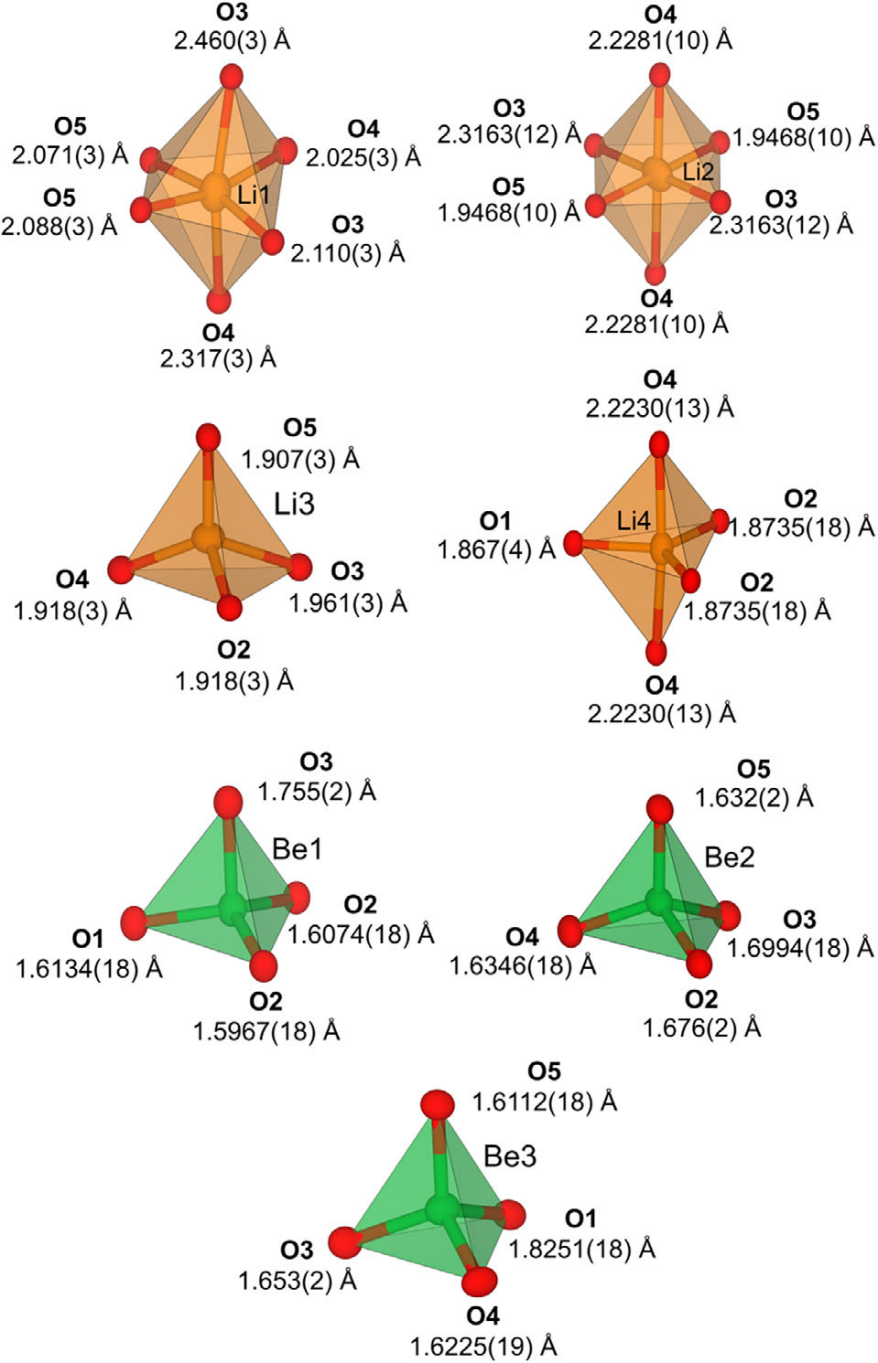
Coordination polyhedra of cations of Li_2_Be_2_O_3_. The displacement ellipsoids are shown with a 90% probability level.

**Figure 3:**
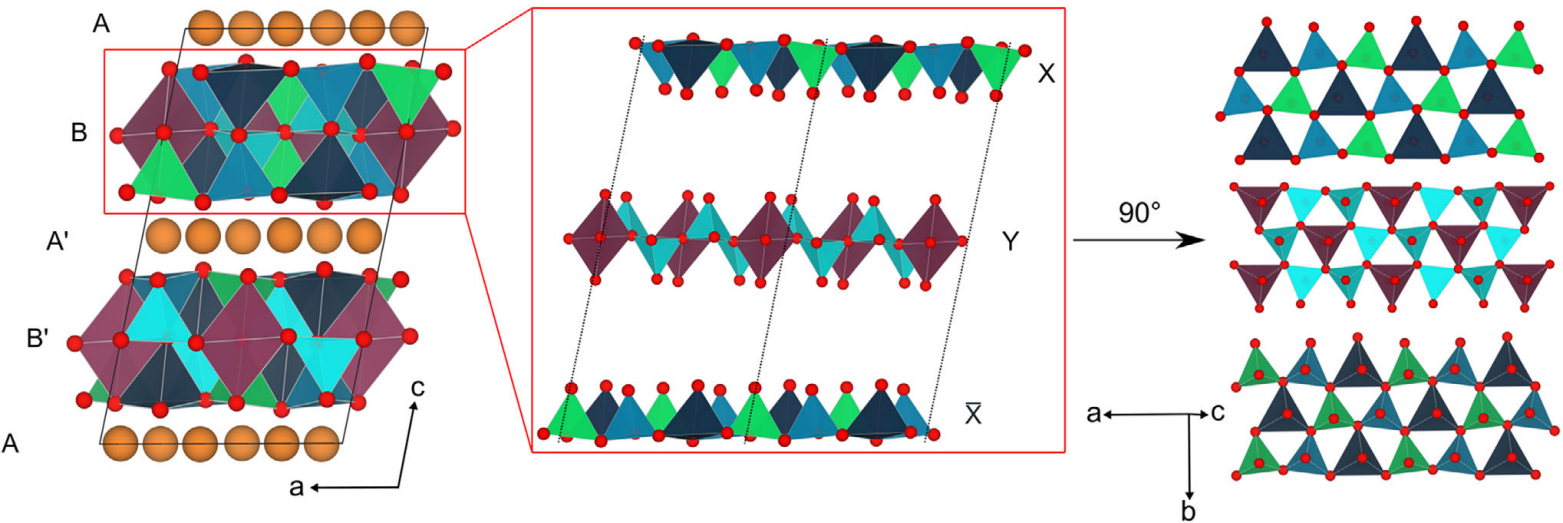
Crystal structure of Li_2_Be_2_O_3_ along [010] (left). The condensed layers can be divided into three sublayers (middle). By rotating 90° around the *b* axis, common structural motifs from wurtzite‐type and megacalsilite‐type compounds become apparent (right). Be1: cyan; Be2: green; Be3: blue; Li1 and 2: orange; Li3: dark blue; Li4: magenta, and O: red.

The refined crystal structure and the coordination polyhedra are illustrated in Figures 2 and 3. The structure consists of infinite layers along [110] of vertex‐and edge‐sharing BeO_4_ and LiO_4_ tetrahedra, as well as LiO_5_ trigonal bipyramids. These layers, stacked along *c*, are separated by Li atoms, which are coordinated as LiO_6_ octahedra. Due to the inversion center, the simple stacking sequence AB is expanded to the sequence ABA'B’ (Figure 3).

For a better understanding of the condensed layers, they can be divided into three sublayers, which in turn contain familiar structural motifs. (Figure 3). The first layer (X) consists of wurtzite‐type vertex‐sharing BeO_4_ (blue and green) and LiO_4_ (dark blue) tetrahedra. The tips of the tetrahedra point all in the opposite *c*‐direction. The intermediate layer (Y) consists solely of vertex‐sharing BeO_4_ tetrahedra, which form *sechser*‐rings (cyan) as defined by Liebau.^[^ ^14^ ^]^ The tips of these tetrahedra point alternately along and against the *c*‐direction.

This structural motif is known from the megacalsilite structure type. In the center of these *sechser*‐rings is a trigonal bipyramidally coordinated Li atom (magenta). The third layer (X̅) is identical with the first one (X), but rotated by 180° around the *b*‐axis so that all tips of the tetrahedra point along the *c‐*axis. These three sublayers are combined as follows to form the complete layer: X and X̅ are assembled in such a way that the tips of the Be2O_4_ tetrahedra (blue) are connected with the ones of the Li3O_4_ tetrahedra (dark blue). The tips of the Be3O_4_ tetrahedra (green) of the X layer are only connected to other Be3O_4_ tetrahedra of the X̅ layer, but not to LiO_4_ tetrahedra. This XX̅ sublayer consists of only vertex‐sharing BeO_4_ and LiO_4_ tetrahedra. By inserting the Y layer, an additional edge sharing between the Be1O_4_ and the Be2O_4_, Be3O_4_ and Li3O_4_ tetrahedra is added. The Li4O_5_ trigonal bipyramid connects the X and X̅ layers via edge sharing additionally. Rotating this composite layer by 90° around the *a*‐axis, a pseudo‐wurtzite‐type structure becomes visible (Figure S3). However, it differs from a wurtzite‐related structure in which the tips of all tetrahedra point in the same direction.

The network topology, which was determined with the TOPOS software, can be described with the point symbol {3^18^.4^24^.5^3^}2{3^27^.4^36^.5^3^}.^[^ ^15^ ^]^ Although the structure consists of building blocks occurring in natural minerals, the topology of this combined network has not been found in any known compound to the best of our knowledge. With respect to the incorporation of Li into the beryllate network, the sum formula can be described as Li[LiBe_2_O_3_], rendering Li_2_Be_2_O_3_ as a lithium lithoberyllate rather than a lithium beryllate.^[^ ^14^ ^]^

Distances and angles of Be–O (1.5967(18)–1.8251(18) Å; 95.06(9)–116.21(10)°) and Li–O tetrahedra (1.907(3)–1.961(3) Å; 90.74(12)–147.83(14)°) as well as Li–O trigonal bipyramids (1.867(4)–2.2230(13) Å) and Li–O octahedra (1.9468(10)–2.460(3) Å) are consistent with those in Li_2_O (1.9962(13) Å; 109.47°), BeO (1.645(3)–1.659(3) Å; 108.84(8)–110.10(8)°) and Li_14_Be_5_B(BO_3_)_9_ (LiO_3_: 1.9160(15) Å; LiO_4_: 1.914(3)–2.010(3) Å; 91.06(11)–132.88(13)°; BeO_4_: 1.594(6)–1.616(4) Å; 107.3(3)–111.8(3)°).^[^ ^16^, ^17^, ^18^ ^]^

The calculation of the Madelung Part of the Lattice Energy (MAPLE) of Li_2_Be_2_O_3_ (14546 kJ mol^−1^) agrees well with the sum of MAPLE values of formally constituting BeO and Li_2_O (14570 kJ mol^−1^, 0.2% difference).^[^ ^19^ ^]^ CHARDI analysis underlines the structural model with effective coordination numbers of 4.60 (Li1 and 2), 3.98 (Li3), and 3.76 (Li4) for Li and 3.80 for Be as well as average total charges of 0.99, 2.00 and, ‐1.96 for Li, Be, and O, respectively.^[^ ^20^ ^]^ Especially for Li4 with an effective coordination number of 3.76, it becomes apparent, that the coordination can also be described by a 3 + 2 coordination, as the axial oxygen atoms have a larger bond distance than the equatorial ones (1.8713 vs. 2.2230 Å, Figure 2). We have decided to describe the coordination polyhedra with a trigonal bipyramid, but the description as a trigonal planar LiO_3_ unit is legitimate, as well. More detailed information about MAPLE and CHARDI calculations can be found in the Supporting Information.

The Rietveld refinement of the powder diffraction pattern (PXRD) of a washed sample of Li_2_Be_2_O_3_, demonstrates the phase‐pure synthesis (Figure S4).

### 2.3 Elemental Analysis

To verify the elemental composition of the found model, a washed sample of Li_2_Be_2_O_3_ was analyzed with inductively coupled plasma optical emission spectrometry (ICP‐OES). The experimentally determined ratio Li:Be of 1.1:1 is consistent with the theoretical value (1:1).

### 2.4 Nuclear Magnetic Resonance (NMR) Spectroscopy

**Figure 4:**
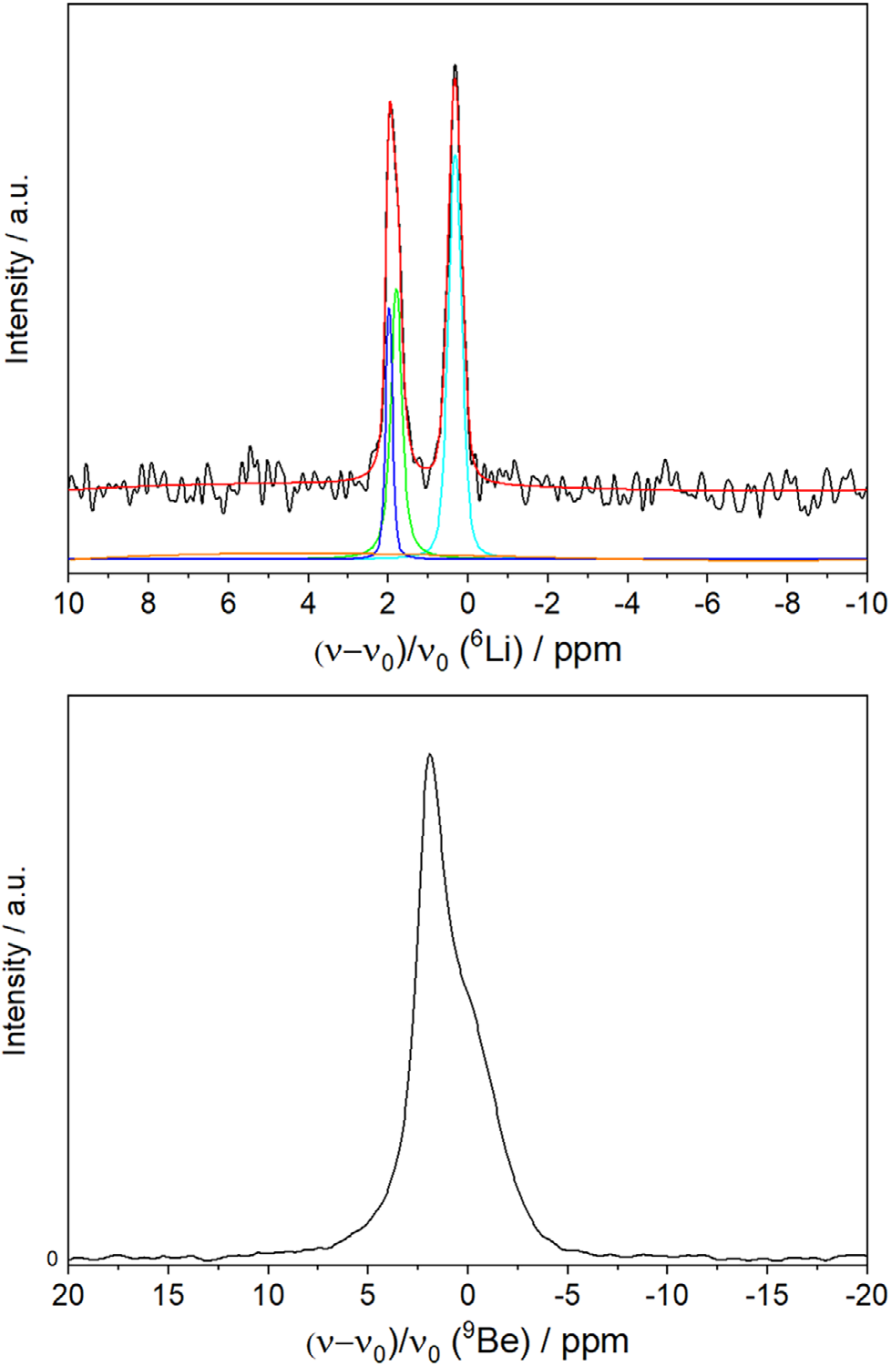
MAS NMR spectra of Li_2_Be_2_O_3_ for ^6^Li (top) and ^9^Be (bottom). The ^6^Li spectrum shows two maxima, which can be deconvoluted into three signals with an integral ratio of 1:1:2, consistent with the four Li sites. The ^9^Be spectrum shows one asymmetric signal, which is likely composed of three signals of the three Be‐sites.

The separation of the condensed Li/Be/O layers by Li atoms raises the question of Li ion conductivity. In order to confirm the structural model and to assess the Li ion conductivity, NMR experiments on ^6^Li, ^7^Li, and ^9^Be were performed with washed Li_2_Be_2_O_3_. The ^6^Li spectrum shows two signals with their maxima at 1.94 and 0.32 ppm (Figure 4). Despite its nuclear spin of 1, quadrupolar coupling effects can be neglected for ^6^Li due to its very small quadrupole moment. A deconvolution of the signals with three functions returns an integral ratio of 1:1:2. The signal at 0.32 ppm with integral 2 can be assigned to the two Li positions, which are both octahedrally coordinated (Li1 and Li2). Accordingly, their respective signals overlap. The other two signals can be associated with Li3 (tetrahedral) and Li4 (trigonal bipyramidal). Although the different coordination polyhedra of the two Li sites would suggest a different shift, CHARDI calculations reveal very similar effective coordination numbers and average bond lengths (average bond length: 1.9259 Å (LiO_4_), 2.0120 Å (LiO_5_); effective coordination number: 3.9838 (LiO_4_); 3.7454 (LiO_5_)). Thus, the resonance frequencies of Li3 and Li4 are nearly identical.

The ^7^Li spectrum shows only one symmetric main signal (Figure S4), which is to be expected for this nucleus in the given crystal structure (detailed information in the Supporting Information).

The width of the spinning sideband pattern in the ^7^Li (*I* = 3/2) spectrum (see Figure S4) indicates the presence of considerable quadrupolar interaction at room temperature, which is not compatible with a marked ion conductivity on the timescale of the NMR measurements. This is in line with the relatively small ellipsoids of the Li atoms refined on the basis of sc‐XRD data.

The ^9^Be spectrum shows a single, asymmetric signal (Figure 4). A deconvolution with three functions of an integral ratio of 1:1:1 is possible and compatible with three evenly occupied crystallographic sites in Li_2_Be_2_O_3_ (Figure S4). Since ^9^Be has a spin (*I* = 3/2), for a more detailed evaluation, the quadrupole moment and its consequences must be taken into account. Using the fitting software DMFIT,^[^ ^21^ ^]^ five free parameters (the chemical shift *δ*_iso_, the quadrupolar coupling constant *C*_q_ and asymmetry parameter *η*_q_, the amplitude and a line broadening factor) are required for each signal of the three Be sites, leading to a total of 15, partially correlated, parameters. Hence, the results are not reliable and vary greatly depending on the selected starting values. Nevertheless, the found ^9^Be signal supports the structural model. More information on NMR measurements can be found in the Supporting Information.

### 2.5 Thermal Stability

The occurrence of different phases in the Li‐Be‐O system, at least in the literature, raises the question of whether there exists a thermodynamically stable phase or if a phase transition is possible. Differential scanning calorimetry (DSC) measurements for Li_2_Be_2_O_3_ show that the compound is stable and does not undergo a phase transition until 1005(2) °C (Figure S5). At this point, the material melts congruently. During cooling, the phase recrystallizes at 995(2) °C. Subsequent PXRD analysis of the recrystallized sample identifies Li_2_Be_2_O_3_ as the only phase. No sign for a phase transition or decomposition into Li_4_BeO_3_ or Li_6_BeO_4_ was observed. The evaluation of the thermal properties of Li_2_Be_2_O_3_ by means of HT powder X‐ray diffraction (PXRD) was not feasible due to the reaction with the quartz capillary at temperatures higher than 400 °C.

### 2.6 UV/VIS Spectroscopy

**Figure 5:**
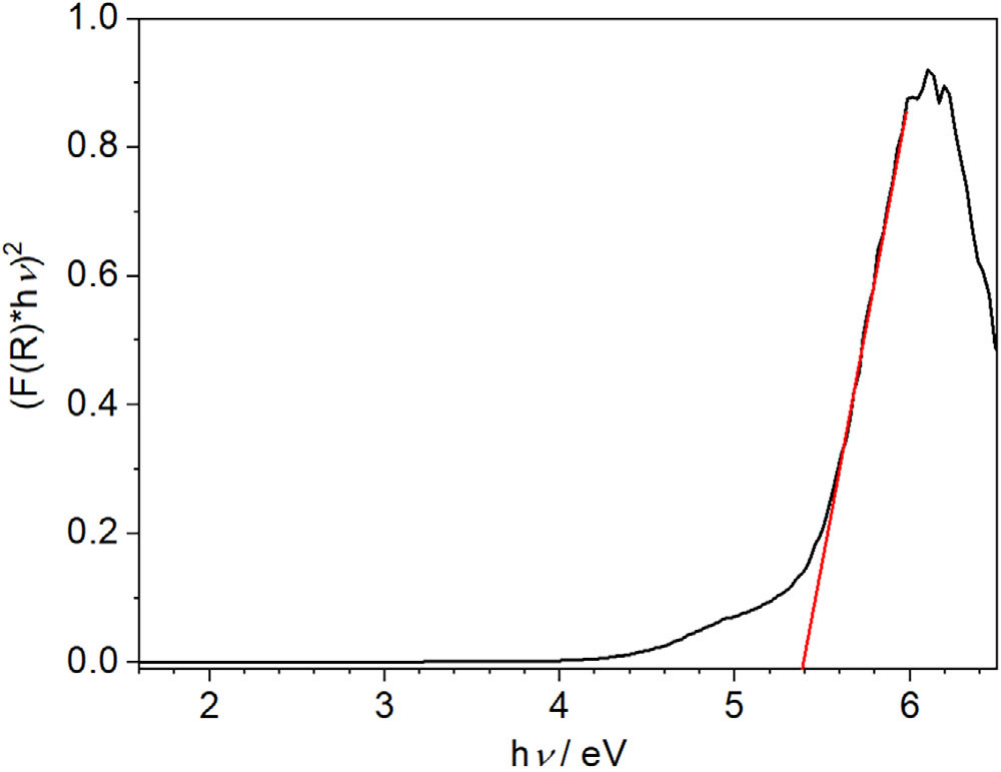
Tauc plot for a washed sample of Li_2_Be_2_O_3_ (black) with a tangent at the inflection point (red).

The optical properties of the title compound were examined by means of UV/Vis spectroscopy from diffuse reflectance spectra using the Kubelka‐Munk function (Figure 5).^[^ ^22^, ^23^ ^]^ Assuming a direct transition, the band gap energy was determined to be ∼5.4 eV, which is in line with the visual impression of colorless, transparent crystals (Figure S1). The Tauc plot shows the presence of an Urbach tail that might arise from defects in the crystal structure.^[^ ^24^ ^]^

### 2.7 Chronoamperometry

For ambient temperature conditions no evidence for ionic conductivity was found by means of NMR spectroscopy or sc‐XRD. As ionic conductivity has been postulated in the literature for sintered Li_2_O·BeO samples at higher temperatures,^[^ ^12^ ^]^ we examined Li_2_Be_2_O_3_ at different temperatures using chronoamperometry. For temperatures below 75 °C, no significant conductivity was detected. At 75 °C, the ionic conductivity was determined to be 1.92(5)∙10^−11^ Scm^−1^ and the electronic conductivity to be 4.9(5)∙10^−12^ Scm^−1^ (Figure S6).^[^ ^25^ ^]^ The obtained results should however, be treated with caution, as they are close to the lower measurement limits. The very low values for both show that Li_2_Be_2_O_3_ is neither an electrical conductor nor an ionic conductor at 75 °C and below. The former is in agreement with the optical impression and the UV/VIS analysis. The chronoamperometric measurement was performed on cold‐pressed pellets of well‐sintered samples (see experimental details). Quenching the samples from the synthesis temperature could increase the defect concentration and thus the ionic conductivity. A more detailed analysis can be found in the Supporting Information.

## 3 Conclusion

Recently, we reported the synthesis of *AE*Li_2_[Be_4_O_6_]:Eu^2+^ (*AE* = Sr, Ba), raising the question of the existence of a ternary lithium beryllium oxide.^[^ ^26^ ^]^ Although the occurrence of multiple phases has been reported in the literature, this study elucidates the structure of the first ternary lithium beryllium oxide. The presented synthesis route for phase‐pure samples of Li_2_Be_2_O_3_ starts from commercially available Li_2_O and BeO and forms the basis for a potential upscaling of the tritium breeder material. The structural model was confirmed by NMR and ICP spectroscopy as well as MAPLE and CHARDI calculations. Electrochemical measurements revealed no measurable ionic or electrical conductivity at 75 °C and below. The latter is in line with the band gap of ∼5.4 eV, which was obtained from diffuse reflectance spectra. The title compound is stable at HT conditions as they occur in a fusion reactor, and melts congruently at 1005(2) °C. Despite extensive experimental studies we could not confirm the existence of Li_4_BeO_3_ and Li_6_BeO_4_, reported by *Kastner* et al. and *Andreev* et al., using our method.^[^ ^10^, ^12^ ^]^ However, the herein presented synthesis and structure elucidation form the basis for future research on the lightest ternary oxide and its potential application as tritium breeding material in the blanket of fusion reactors.

## 4 Experimental Section

### Safety notice

Beryllium and its compounds can cause different diseases like acute berylliosis, chronic beryllium disease (CBD), contact dermatitis, and cancer. Especially the inhalation of Be‐containing dust or the contact with soluble Be salts is dangerous. Consequently, the complete handling of starting materials and Be‐containing samples was carried out in a designated glovebox. Samples outside the glovebox were only transported in closed vessels. For unavoidable work with Be outside the glovebox (e.g., cleaning working equipment) care must be taken to additional safety precautions like FFP3‐mask and the avoidance of dusts. Guidelines on how to work with Be are available in the literature.^[^ ^27^, ^28^ ^]^

### HT synthesis

Li_2_Be_2_O_3_ was synthesized in a HT approach, starting from stoichiometric amounts of Li_2_O (Alfa Aesar, 99.5%) and BeO (Alfa Aesar, 99.95%) under an Ar atmosphere. An excess of 10% Li_2_O was additionally added. Because of safety reasons (see above), the starting materials were handled in an Ar‐filled glovebox (Unilab, MBraun, Garching; O_2_, H_2_O < 1 ppm). The starting materials were finely ground and pressed to a thin pill with a diameter of 10 mm. This pill was placed in a tungsten crucible, which was transferred to a radiofrequency furnace (8TIG 10/100; Hüttinger Elektronik Freiburg, Germany). The crucible with the pill inside was heated to 1500 °C within 10 minutes, held at this temperature for 10 minutes, cooled down to 1000 °C in 10 minutes and held at this temperature for 5 hours, cooled down to 500 °C within 2 hours, closed by switching off the furnace and natural cooling. The short dwelling step at 1500 °C was implemented to melt Li_2_O, what lead to a better homogeneity of the sample and bigger crystals. Although, the sample is only held for 10 minutes at this temperature, Li_2_O starts to evaporate. For this, the excess for 10% was added. To remove residual Li_2_O, the sample was washed with distilled water after the synthesis. Detailed information on the design of the radiofrequency furnace is available in the literature.^[^ ^29^ ^]^

### sc‐XRD, structure solution, and refinement

sc‐XRD measurements were performed on selected crystals that showed extinction under linear polarized light. The selected crystals were measured by combined ϕ‐ and ω‐scans on a Bruker D8 Venture TXS diffractometer with a rotating anode (Mo‐K*α*_1_ radiation, *λ*  =  0.71073 Å) and multilayer monochromator. Indexing, integration, absorption correction, and the space group determination were performed with the APEX3 software package.^[^ ^30^ ^]^ The structure was solved using SHELXT.^[^ ^31^ ^]^ The structural model was refined by the least squares method using SHELXL.^[^ ^32^ ^]^ The structure was visualized using VESTA.^[^ ^33^ ^]^

### PXRD and Rietveld refinement

For PXRD measurements the sample was ground and sealed in a glass capillary (0.3 mm outer diameter, Hilgendberg, Malsfeld). After centering the capillary on a rotating goniometer head, data was collected on a STOE Stadi P diffractometer (STOE and Cie GmbH, Darmstadt) in a modified Debye‐Scherrer geometry with Cu‐K*α*_1_ radiation (*λ*  =  1.54060 Å) equipped with a MYTHEN 1 K strip detector and a Ge(111) monochromator. The Rietveld refinement of PXRD data of Li_2_Be_2_O_3_ was carried out on the structural model received from sc‐XRD data to demonstrate a phase‐pure synthesis. For the refinement of the program package TOPAS Academic was used.^[^ ^34^, ^35^ ^]^ The peak profiles were described with the fundamental parameter approach. A potential preferred orientation of the crystallites was accounted for with a fourth order harmonic function, and the background was modeled by a shifted Chebyshev polynomial.^[^ ^36^, ^37^ ^]^ The result was plotted using Origin.^[^ ^38^ ^]^

### Inductively coupled plasma optical emission spectrometry (ICP‐OES)

Elemental analysis was conducted at a Varian Vista RL with a 40 MHz RF generator and a VistaChip CCD detector. A washed sample of Li_2_Be_2_O_3_ was dissolved in a mixture of *aqua regia* and HF. The use of HF is essential.

### Scanning electron microscopy (SEM) and energy dispersive X‐ray (EDX) spectroscopy

For electron microscope investigations, the sample was positioned on a self‐adhesive carbon foil and coated with carbon using an electron beam evaporator (BAL‐TEC MED 020, BalTec AG, Pfäffikon) to guarantee electrical conductivity. For SEM imaging and EDX measurements, a Dualbeam Helios Nanolab G3 UC (FEI, Hilsboro) equipped with an X‐Max 80 SDD detector (Oxford instruments, Abingdon) was used. The data was processed with the Aztec software.^[^ ^39^ ^]^ The accelerating voltage for SEM imaging and EDX measurements was 5.0 kV.

### Solid‐state magic‐angle spinning (MAS) NMR spectroscopy

For solid‐state NMR measurements, the sample was ground and loaded in a ZrO_2_ rotor with an outer diameter of 2.5 mm. Tightly packed Teflon tape was used as a spacer at the bottom and the top of the rotor to avoid direct contact of the Be‐containing sample with the environment. NMR spectra were collected at 10 kHz spinning frequency on an Avance III 500 spectrometer (Bruker, Karlsruhe) with an 11.7 T magnet (500.25 MHz ^1^H frequency). Spectra were fitted using Igor Pro 7.^[^ ^40^ ^]^ All spectra were indirectly referenced to ^1^H in 100% tetramethylsilane (TMS) at − 0.1240 ppm.

### Thermal Analysis

The thermal behavior was examined by DSC. A sample of Li_2_Be_2_O_3_ (8.5 mg) was filled in a small tantalum ampule, which was sealed by arc melting under an argon atmosphere. DSC data of subsequent heating and cooling cycles to 1100 °C with 5 °C/min were collected under argon flow using a Jupiter STA 449 F5 setup (Netzsch GmbH, Selb, Germany). Data were processed with the Proteus software package.^[^ ^41^ ^]^ A reference measurement was conducted with an argon‐filled tantalum ampule with similar dimensions to those of the sample crucible.

### UV/VIS reflectance spectroscopy

The optical band gap was assessed via UV/VIS spectroscopy. Diffuse reflectance spectra were collected at room temperature on a Jasco V‐650 UV/VIS spectrophotometer equipped with a Czerny–Turner mount, a photomultiplier tube detector, and deuterium (190–350 nm) and halogen (330–900 nm) lamps as light sources. The measured reflectance spectra were converted to pseudo‐absorption spectra using the Kubelka‐Munk function (*F(R) = (1 − R)* *2* */2R*).^[^ ^22^ ^]^ The results for (*F(R)hν*)^1/^ *^n^* (with *n *= 1/2, assuming a direct band gap) are plotted versus *hν* in a so‐called Tauc‐Plot.^[^ ^23^ ^]^ The tangent that touches the inflection point indicates the band gap energy.

### Electrochemical measurements

For the measurement of electrochemical impedance spectroscopy as well as chronoamperometry, the sample was compacted by uniaxial cold pressing (1.5 t, 2081 MPa) into a pellet with a diameter of 3 mm and a thickness of 0.184 mm. The measurements were carried out using an RHD Instruments TSC SW closed impedance cell in a two‐electrode setup (stainless steel electrodes/ ion blocking configuration) under an argon atmosphere. The pressure during the measurement was about 2.2 MPa. The chronoamperometry measurements were carried out with an Ivium compactstat.h (24‐bit device) at 0, 1, 2, 3, and 3.5 V. A data point was recorded every 0.2 seconds.

## Supporting Information

The authors have cited additional references within the Supporting Information.

## Conflict of Interest

The authors declare no conflict of interests.

## Supporting information



Supplementary Information

## Data Availability

The data that support the findings of this study are available in the supplementary material of this article.
