# Supplementary material for: Long Used but Hardly Known: Synthesis and Crystal Structure of Tritium Breeding Li2Be2O3
Source: Chemistry. 2025 Aug 11;31(47):e02209. doi: 10.1002/chem.202502209 (PMC12376256; doi:10.1002/chem.202502209)
Supplement: Supplementary file 1 — Supplementary Information [file CHEM-31-e02209-s001.pdf]

SUPPORTING INFORMATION

Long Used But Hardly Known: Synthesis and Crystal Structure of Tritium Breeding $Li_2Be_2O_3$

Georg Krach,[a] Jennifer Steinadler,[a] Robert Calaminus,[a,b] Bettina V. Lotsch,[a,b] and Wolfgang Schnick*[a]

[a] Dr. G. Krach, J. Steinadler, K. Witthaut, R. Calaminus, Prof. Dr. B. V. Lotsch, Prof. Dr. W. Schnick
Department of Chemistry, University of Munich (LMU)
Butenandtstraße 5–13, 81377 Munich (Germany)
E-mail: wolfgang.schnick@uni-muenchen.de
[b] R. Calaminus, Prof. Dr. B. V. Lotsch
Max Planck Institute for Solid State Research
Heisenbergstraße 1, 70569 Stuttgart (Germany)

SUPPORTING INFORMATION

Table of Contents

Results and Discussion **3**
SEM images and ICP measurements 3
Rietveld refinement 4
Crystallographic data 5
Charge distribution (CHARDI) 7
MAPLE 7
Solid state MAS NMR spectroscopy 8
DSC measurements 8
Electrochemical measurements 9

Results and Discussion

SEM images and ICP measurements

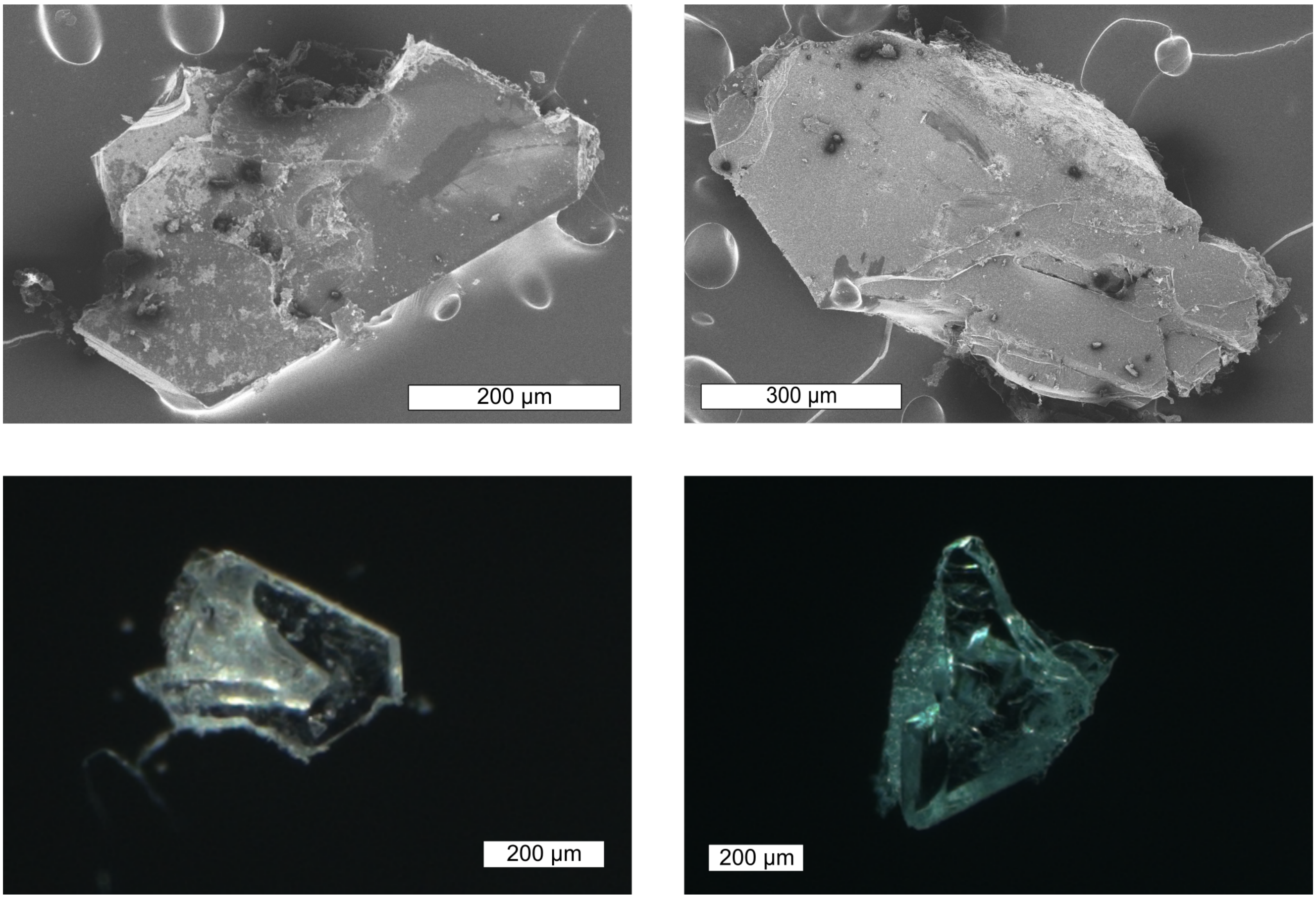

Figure S1. Images of crystals of $Li_2Be_2O_3$ taken in the SEM (a, b) and under the light microscope (c, d). The crystals are up to 400 µm in length and 200 µm in width. The colorless and transparent properties become apparent.

EDX measurements were carried out on different crystallites of $Li_2Be_2O_3$. No other elements than Be and O were detected. Li cannot be detected by means of EDX. Hence, for the precise determination of the Li:Be ratio, ICP measurements were conducted. A sample of washed $Li_2Be_2O_3$ was solved in mixture of *aqua regia* and HF. The ratio of Li:Be was measured by means of a double determination and is in line with theoretical value (exp.: 1.1:1, theor. 1:1).

Rietveld refinement

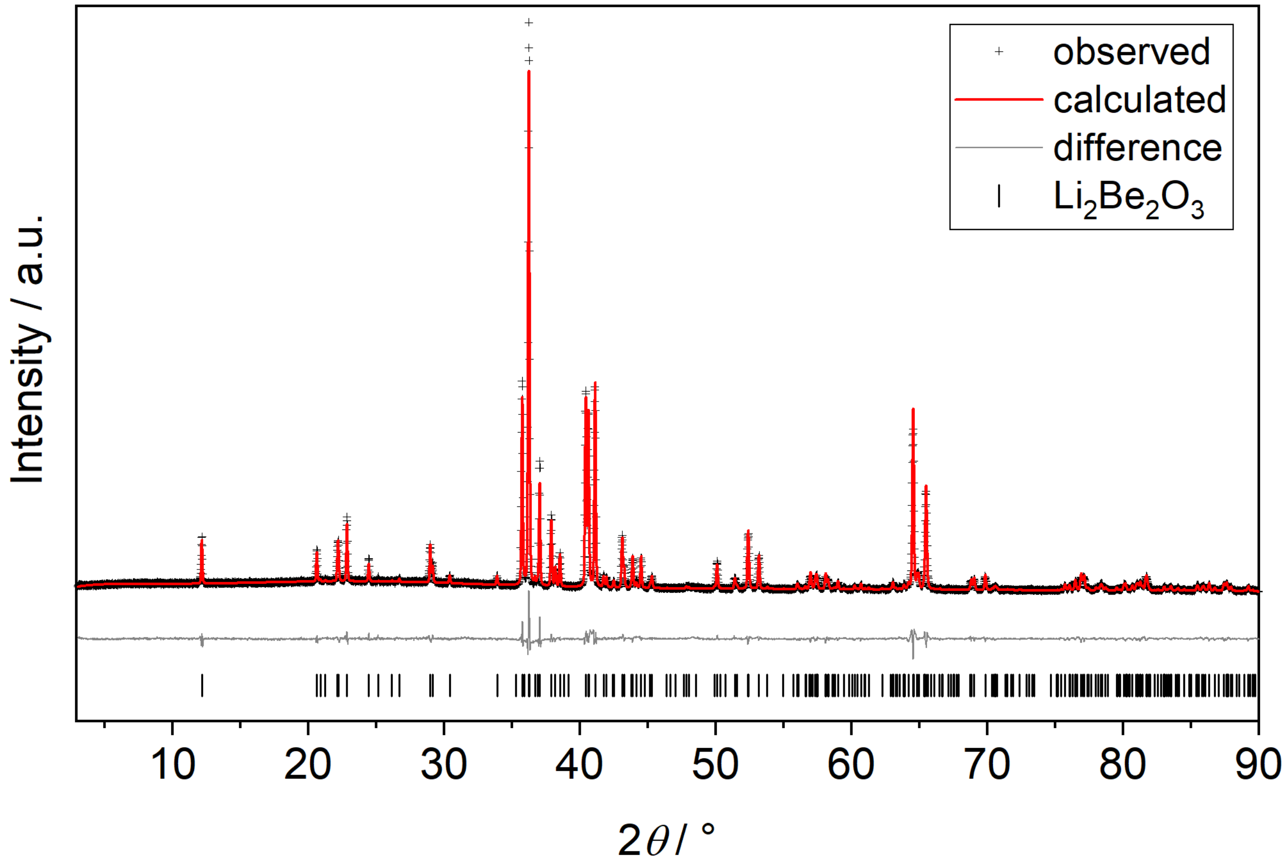

Figure S2. Result of the Rietveld refinement of $Li_2Be_2O_3$. Observed (black crosses), calculated (red line) powder X-ray diffraction patterns (CuKα_1, λ = 1.540596 Å) and difference plot (gray line). Positions of Bragg reflections of $Li_2Be_2O_3$ are given as black bars.

SUPPORTING INFORMATION

Crystallographic data

Table S1. Crystallographic data of $Li_2Be_2O_3$. Standard deviations are given in parenthesis.

**Formula**	**$Li_2Be_2O_3$**
Crystal system	monoclinic
Space group	*C*2/*c* (no. 15)
Lattice parameters / Å, °	*a* = 8.547(2)
	b = 5.0224(14)
	c = 14.875(6)
	β = 101.896(13)
Cell volume /$Å^3$	624.8(3)
Formula units per unit cell	12
Density / g cm^{-3}	2.548
Molecular weight / g mol^{-1}	79.9
Linear absorption coefficient / mm^{-1}	0.211
T_{min}/T_{max}	0.839
Diffractometer	D8 Venture
Radiation	Mo-Kα_1 (λ = 0.71973 Å)
Absorption correction	multi-scan
F(000)	456
θ range / °	4.734 ≤ θ ≤ 30.502
Total no. of reflections	3606
Independent reflections [$I \geq 2\sigma(I)$/all]	801/948
R_{int}	0.043
Refined parameters	98
Goodness of fit	1.103
R-values [$I \geq 2\sigma(I)$]	R_1 = 0.0376; *wR*2 = 0.0857
R-values [all data]	R_1 = 0.0466; *wR*2 = 0.0892
$\Delta\rho_{max}$, $\Delta\rho_{min}$ / e $Å^3$	0.349; –0.231

Table S2. Wyckoff positions, coordinates, isotropic thermal displacement parameters and occupancy of $Li_2Be_2O_3$. Standard deviations are given in parenthesis.

Atom	Wyck.	*x*	*y*	*z*	U_{eq} / $Å^2$	Occ.
Li1	8*f*	0.4143(3)	0.2414(5)	0.50131(16)	0.0212(8)	1
Li2	4*c*	3/4	1/4	1/2	0.0224(10)	1
Li3	8*f*	0.6992(3)	0.9126(4)	0.37272(16)	0.0143(6)	1
Li4	4*e*	1/2	0.1065(6)	1/4	0.0151(9)	1
Be1	8*f*	0.65936(18)	0.6078(3)	0.22619(11)	0.0103(4)	1
Be2	8*f*	0.86565(18)	0.4474(3)	0.37195(11)	0.0100(4)	1
Be3	8*f*	0.54782(19)	0.4323(3)	0.37437(11)	0.0107(4)	1
O1	4*e*	1/2	0.4782(2)	1/4	0.0096(3)	1
O2	8*f*	0.81254(10)	0.42203(17)	0.25738(6)	0.0090(2)	1
O3	8*f*	0.39864(10)	0.61789(17)	0.39402(6)	0.0101(3)	1
O4	8*f*	0.52912(10)	0.12304(17)	0.40189(6)	0.0106(3)	1
O5	8*f*	0.72050(9)	0.55660(17)	0.41817(6)	0.0098(2)	1

Table S3. Anisotropic displacement parameters (U_{ij} / Å^2) of $Li_2Be_2O_3$ derived from single-crystal data. Standard deviations are given in parentheses.

Atom	U_{11} / Å^2	U_{22} / Å^2	U_{33} / Å^2	U_{12} / Å^2	U_{13} / Å^2	U_{23} / Å^2
Li1	0.0160(13)	0.0266(13)	0.0207(13)	–0.0016(9)	0.0032(10)	–0.0067(10)
Li2	0.0258(19)	0.0164(16)	0.0237(18)	0.0023(13)	0.0019(14)	0.0070(14)
Li3	0.0149(11)	0.0118(10)	0.0163(11)	0.0003(8)	0.0038(8)	0.0009(8)
Li4	0.0136(15)	0.0098(14)	0.0225(17)	0	0.0050(12)	0
Be1	0.0094(7)	0.0074(7)	0.0141(8)	–0.0003(5)	0.0022(6)	0.0000(5)
Be2	0.0095(7)	0.0082(7)	0.0127(7)	0.0001(5)	0.0030(5)	0.0001(6)
Be3	0.0097(7)	0.0092(7)	0.0131(8)	–0.0003(5)	0.0019(5)	–0.0010(6)
O1	0.0082(6)	0.0079(6)	0.0131(6)	0	0.0029(4)	0
O2	0.0086(4)	0.0069(4)	0.0116(4)	0.0003(3)	0.0021(3)	0.0004(3)
O3	0.0092(4)	0.0088(4)	0.0125(5)	0.0002(3)	0.0025(3)	0.0000(3)
O4	0.0099(4)	0.0076(4)	0.0141(5)	–0.0009(3)	0.0023(3)	–0.0004(3)
O5	0.0081(4)	0.0086(4)	0.0128(4)	–0.0001(3)	0.0023(3)	–0.0002(3)

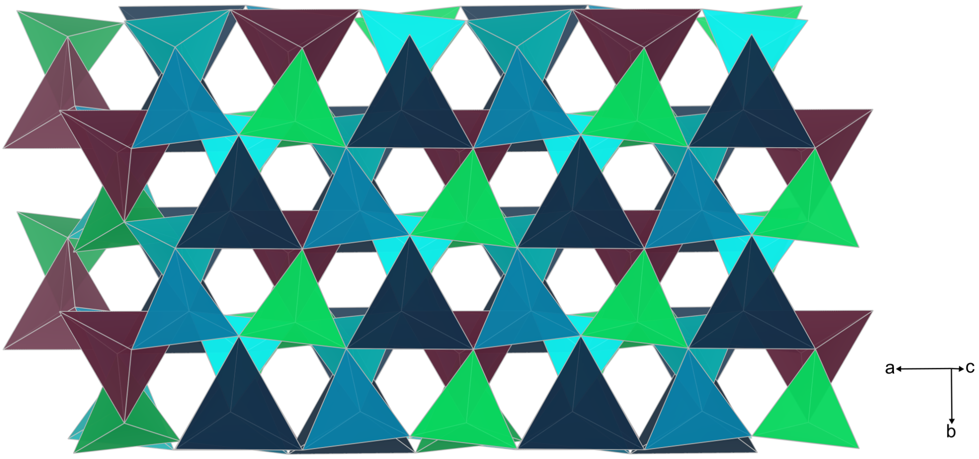

Figure S3. Crystal structure of $Li_2Be_2O_3$ viewed approximately along the *c*-axis. A wurtzite-type structure becomes apparent. However, it differs from a wurtzite structure in which the tips of all tetrahedra point in the same direction. LiO_6 octahedra (Li1 and Li2) are not shown. Be1: cyan; Be2: green; Be3: blue; Li3: dark blue; Li4: magenta

SUPPORTING INFORMATION

Charge distribution (CHARDI)

Table S4: Results of the CHARDI analysis for $Li_2Be_2O_3$. All effective coordination numbers and the mean total charges are in good agreement with the theoretical values.

**Polyhedron**	**Li1 O3 O3 O4 O4 O5 O5**	**Li2 O3 O3 O4 O4 O5 O5**	**Li3 O2 O3 O4 O5**
Average bong length / Å	2.1788	2.1637	1.9259
Polyhedral volume / Å^3	13.2131	12.9920	3.5378
Distortion index (bond length)	0.06426	0.06685	0.00917
Quadratic elongation	1.0342	1.0317	1.0242
Bond angle variance / °^{2}	87.8732	72.9841	88.7079
Effective coordination number (theory)	4.9568 (6)	4.2365 (6)	3.9838 (4)
**Total charge (theory)**			
Li	1.007 (1)	0.980 (1)	0.995 (1)
O1			
O2			-2.172 (-2)
O3	-1.801 (-2)	-1.801 (-2)	-1.801 (-2)
O4	-1.917 (-2)	-1.917 (-2)	-1.917 (-2)
O5	-2.171 (-2)	-2.171 (-2)	-2.171 (-2)

**Polyhedron**	**Li4 O1 O2 O2 O4 O4**	**Be1 O1 O2 O2 O3**	**Be1 O2 O3 O4 O5**	**Be3 O1 O3 O4 O5**
Average bong length / Å	2.0120	1.6432	1.6605	1.6781
Polyhedral volume / Å^3	6.6462	2.2148	2.3176	2.3808
Distortion index (bond length)	0.08391	0.03405	0.01628	0.04381
Quadratic elongation	-	1.0202	1.0095	1.0150
Bond angle variance / °^{2}	-	75.4682	37.0843	49.1942
Effective coordination number (theory)	3.7564 (3+2)	3.7887 (4)	3.9578 (4)	3.6454 (4)
**Total charge (theory)**				
Li / Be	0.977 (1)	1.984 (2)	1.991 (2)	2.054 (2)
O1	-1.878 (-2)	-1.878 (-2)		-1.878 (-2)
O2	-2.172 (-2)	-2.172 (-2)	-2.172 (-2)	
O3		-1.768 (-2)	-1.768 (-2)	-1.768 (-2)
O4	-1.931 (-2)		-1.931 (-2)	-1.931 (-2)
O5			-2.190 (-2)	-2.190 (-2)

MAPLE

Table S5: Results of MAPLE calculations show a difference of 0.2% in the Madelung part of lattice energy for $Li_2Be_2O_3$ compared to the sum of the respective binary oxides Li_2O and BeO.

	Li_2O + 2 BeO	→	$Li_2Be_2O_3$	
Li_2O	3506 kJ/mol	$Li_2Be_2O_3$	14546 kJ/mol	
BeO	2 x 5532 kJ/mol			
	14570 kJ / mol		14546 kJ / mol	**0.2% difference**

Solid state MAS NMR spectroscopy

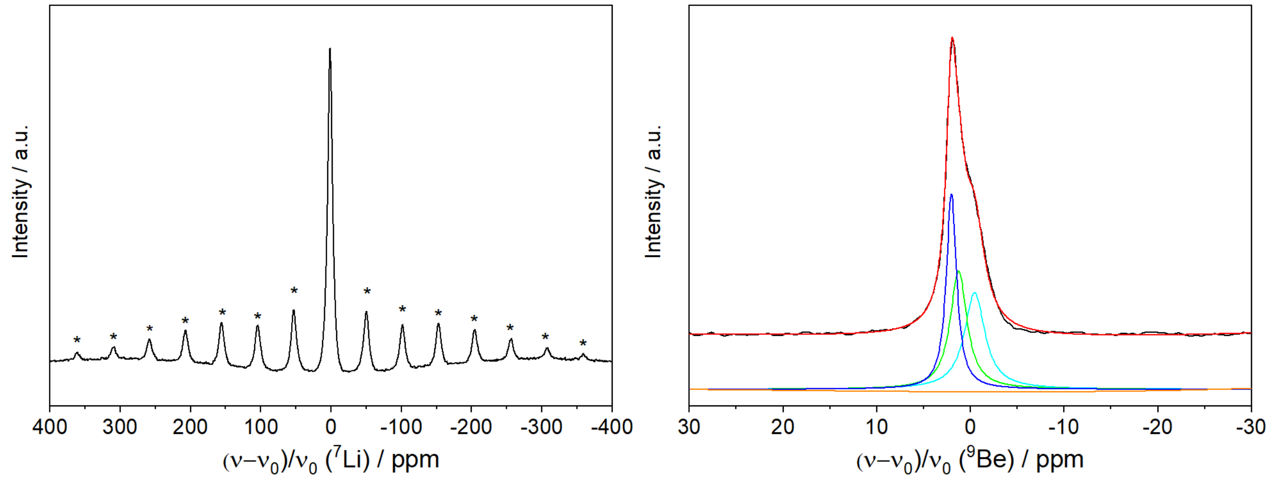

Figure S4: Solid state MAS NMR spectra of ^{7}Li (left) and ^{9}Be (right) of $Li_2Be_2O_3$ each at a spinning frequency of 10 kHz. Spinning side-bands are marked with asterisks. The ^{7}Li spectrum shows a single resonance. For more information see below. The deconvolution of the ^{9}Be spectrum is possible with three functions. However, a more detailed evaluation is not meaningful as depicted in the main manuscript.

Detailed information for ^{7}Li spectrum: The resonance frequencies of the central transitions (CTs) of this spin $I = 3/2$ nucleus are only affected by small second-order effects of the quadrupolar interaction and the chemical shift whose range is very moderate for this light element. Furthermore, due to its larger quadrupole moment and residual homonuclear dipolar couplings the obtained signals are significantly broader compared to ^{6}Li and an overlap of the CTs corresponding to the four Li sites is to be expected.

DSC measurements

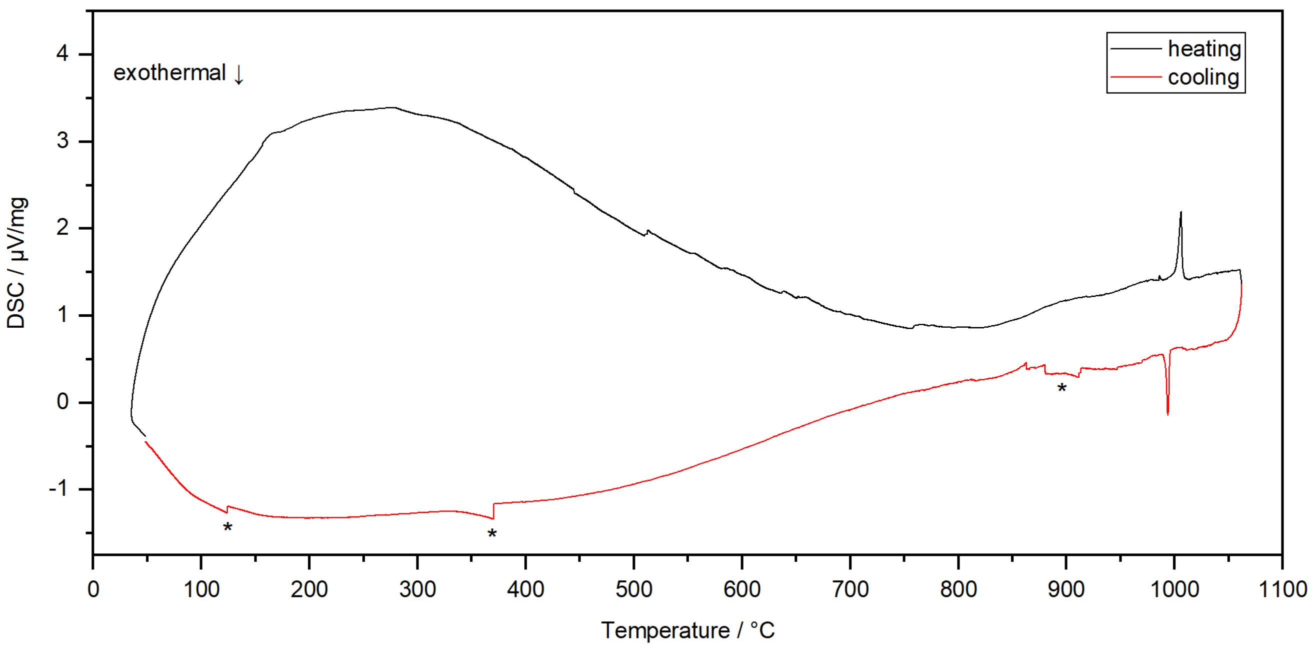

Figure S5. Differential thermal analysis of $Li_2Be_2O_3$ with heating (black) and cooling (red). The endothermic melting signal at 1005(2) °C and the exothermic signal of recrystallization at 995(2)°C are clearly visible. Measurement artefacts are marked with an asterisk.

Electrochemical measurements

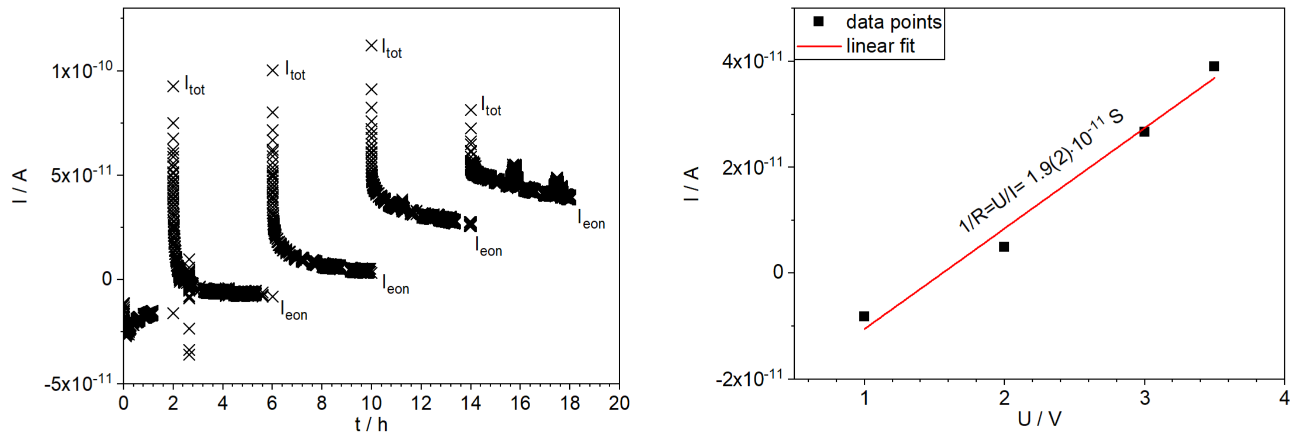

Figure S6. Chronoamperometry measurement at 75°C (left), where the potential was increased to 1, 2, 3 and 3.5 V after an initial open-circuit phase (U=0 V) after every 4 hours. The data points at the end of each 4-hour step were plotted in an I-V diagram (right). The electronic resistance can be derived from the linear fit of these data points.

Ionic and electronic conductivities were derived from the chronoamperometry measurement shown in Figure S6. The electronic conductivity can be determined by plotting the steady-state current after each potential step (see I_{eon} markings in Figure S6) in an I-V plot. A linear fit of those points (Ohm's law) results in the reciprocal electronic resistance ($1/R_{eon,75°C}$=1.9(2)·10^{-12} S, $R_{eon,75°C}$ =5.27·10^{10} Ω). Using Equation S1, where d is the pellet thickness, A is the pellet area and R is the resistance, this leads to an electronic conductivity of $\sigma_{eon,75°C}$= 4.9(5)·10^{-12} Scm^{-1}. However, the steady state was not always reached after 4 hours, so the calculated value represents the upper limit of the electronic conductivity.

$$\sigma = \frac{1}{R}\frac{d}{A} \text{ (S1)}$$

The total resistance can be derived from the first current value after a new potential is applied (see I_{tot} marking) and before the subsequent drop. Since the underlying process is extremely fast, even the interval between the measuring points every 0.2 s is not sufficient to capture the total resistance perfectly. Therefore, the highest current value was used to determine the total resistance and yet the resulting total conductivity only marks the lower limit. Applying Ohm's law results in a total resistance of $R_{tot,75°C}$ =1.08·10^{10} Ω. Using Equation S1, a total conductivity of $\sigma_{tot,75°C}$= 2.4·10^{-11} Scm^{-1} is obtained. From this value, the ionic conductivity can be calculated by Equation S2 as $\sigma_{ion,75°C}$= 1.92(5)·10^{-11} Scm^{-1} ($R_{ion,75°C}$ = 1.36·10^{10} Ω).

$$\sigma_{ion} = \sigma_{tot} - \sigma_{eon} \text{ (S2)}$$
